# A Transcription Factor-Centric View of Transcription Regulation

**DOI:** 10.3390/biology15100769

**Published:** 2026-05-12

**Authors:** Jing He, Jiahui Chi, Siqian Feng

**Affiliations:** Key Laboratory of Developmental Genes and Human Disease, School of Life Science and Technology, Southeast University, Nanjing 210032, China

**Keywords:** transcription, transcription factor, enhancer, promoter, DNA loop, condensate, Mediator

## Abstract

Transcription is arguably the most critical regulatory step in gene expression. In eukaryotes, a promoter is necessary for transcription, but it alone often does not lead to significant gene transcription. Distal regulatory elements called enhancers can greatly increase transcription levels. Sequence-specific transcription factors recruited to the enhancers play pivot roles in regulating promoter activities. These enhancer-bound transcription factors represent a central node in biological regulation, by converting various internal and external regulatory signals into biological responses in the form of target gene transcription. In this review, we take a transcription factor-centric view, and summarize recent advances in our understanding of how transcription factors regulate promoter activities. We also highlight significant outstanding questions in the field, and propose possible strategies to solve these questions.

## Simple Summary

Transcription is probably the most important step in gene regulation, and transcription factors play central roles in transcription regulation. Sequence-specific transcription factors bound to enhancers regulate promoter activities and stimulate transcription levels. In this article, we comprehensively review how transcription factors bind to their target enhancers, how they regulate promoter activities once bound to enhancers, and how enhancers locate the appropriate promoters in crowded nuclei. We also point out significant unsolved questions in the transcription regulation field, and propose potential paths to solve these questions.

## 1. Introduction

Gene regulation is at the heart of all biological systems. In metazoans, the vast majority of cells contain the same genome, but they differentiate into many different cell types with distinct functions. The reason behind cell fate determination is differential gene regulation: each cell type only expresses the set of genes specific to that cell type, while repressing the genes characteristic of other cell types. Gene expression is a multistep process. Transcription, the process of synthesizing RNA based on base pairing with genomic DNA, is the first step in gene expression, and is arguably the most critical step of gene regulation. Eukaryotic transcription is carried out by three different RNA polymerases [1]. This review mainly focuses on the transcription of protein-coding genes, all of which are transcribed by RNA polymerase II (RNA pol II).

**Figure 1:**
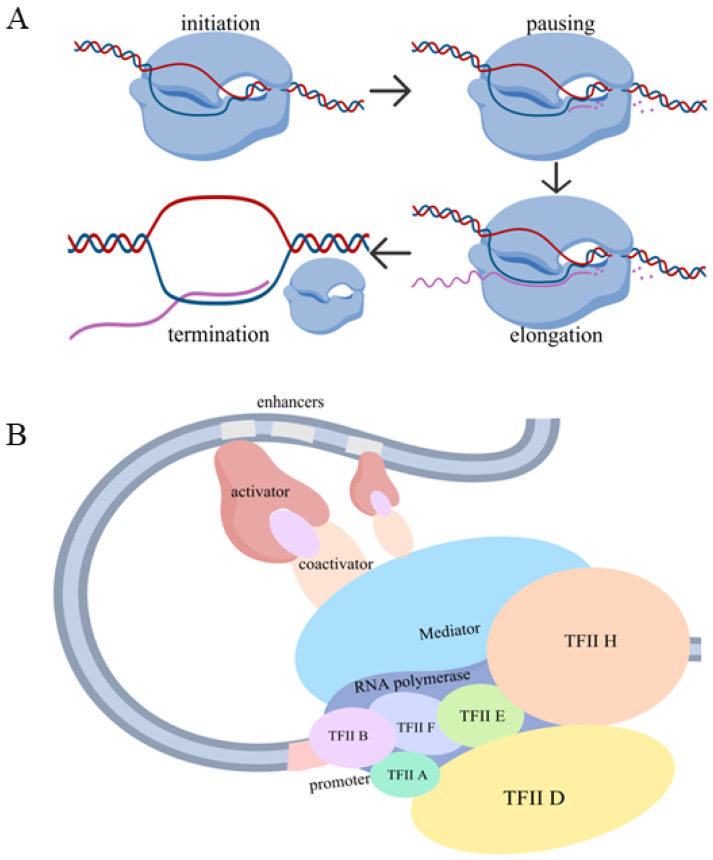
Overview of eukaryotic transcription. (**A**) Schematic showing major steps of eukaryotic transcription, including initiation, promoter-proximal pausing, pause release, productive elongation and termination. (**B**) Schematic showing major cis-regulatory elements and trans-factors that regulate eukaryotic transcription. Promoters are where RNA polymerase II initiates transcription, and are bound by general transcription factors (GTFs), such as TFIIA, TFIIB, TFIID, TFIIE, TFIIF and TFIIH. Promoters are necessary for transcription, but often do not sustain high levels of transcription. Enhancers are distal cis-regulatory elements to which sequence-specific transcription factors are bound. Enhancers significantly boost transcription levels, and are responsible for tissue- and stage-specific transcription of various genes. Mediator complex plays important roles in connecting enhancers and the promoters they regulate.

Transcription by RNA pol II is an integrated process involving multiple steps: initiation [2], promoter-proximal pausing and pause release [3], productive elongation [4] and termination [5]. Each of these steps, as well as RNA splicing and degradation, represents a potential regulatory point. The current results point to transcription initiation and pause release as the most critical regulatory points [6]. Initiation is the process by which RNA pol II molecules start the de novo synthesis of nascent RNA molecules. Traditionally, it was believed that once an RNA pol II molecule initiated transcription, it usually finished the entire transcription cycle. However, research in the past two decades has shown that for many eukaryotic genes, especially developmental genes and signaling genes, RNA pol II molecules pause on DNA after transcribing 50–70 nucleotides of RNA, until they receive another signal that releases them from the paused state and initiates the productive elongation phase [3] (Figure 1A).

In eukaryotes, transcription is regulated by cis-regulatory elements, such as promoters and enhancers, as well as trans-acting factors (proteins and RNAs) that interact with various cis-regulatory elements. The genomic region encompassing the transcription start site (TSS) is the promoter, at which point RNA pol II initiates transcription [7,8]. RNA pol II does not initiate transcription on its own. Rather, transcription initiation requires the formation of a Preinitiation Complex (PIC) at the promoter [2]. The PIC contains general transcription factors (GTFs), such as TFIIA, TFIIB, TFIID, TFIIE, TFIIF and TFIIH, and is stabilized by the Mediator complex [9,10]. While promoter-proximal elements can be sufficient for the transcription of some housekeeping genes [11], most genes whose transcription varies across tissues, developmental stages, or environmental conditions require the promoter to act together with additional regulatory elements—such as enhancers—to achieve appropriate and robust transcription. In addition, high levels of transcription require distal cis-regulatory elements called enhancers, which significantly boost the transcription level in a tissue-specific and developmental stage-specific manner [12,13,14]. Enhancers are also responsible for transcription responses to internal signals such as hormones, and external signals like environmental stresses [15]. Within enhancers, there are binding sites for sequence-specific transcription factors (hereafter transcription factors, or TFs) [16]. TFs bind to enhancers and can stimulate transcription levels at the promoters [16]. The Mediator complex is a large multi-subunit complex and is an important bridge between enhancer-bound TFs and promoters [9,10]. Many TFs have been shown to physically interact with different Mediator subunits [9,10] (Figure 1B). If the chromatin where a gene resides is in a closed conformation, the chromatin must be remodeled so the various proteins regulating transcription can have access [17]. Factors like chromatin remodelers and pioneer factors play important roles in this chromatin remodeling process [18,19,20,21,22].

In this review, we focus on sequence-specific transcription factors and review how they perform their regulatory functions in vivo. The action of TFs can conceptually be divided into two phases, TF-DNA binding and TF action on promoter activity. How TFs bind to DNA is currently understood significantly better than how TFs regulate promoter activities once bound to enhancers. In the following sections, we review the following aspects of TF functions in vivo. We first give a comprehensive summary of the mechanisms by which TFs precisely find and bind to their target sites in vivo. We then review how TFs regulate promoter activities once they bind to their targets in enhancers. Finally, we provide a brief review of how enhancers find the correct promoters they should regulate. We also outline outstanding questions and challenges in the field, and point out what strategies could be taken to tackle these challenges.

## 2. Transcription Factor–DNA Binding In Vivo

For any sequence-specific DNA-binding protein like a TF, the task of finding its target sites within the nuclear genome is anything but trivial. Earlier studies, many of which focused on type II restriction enzymes, identified sliding (also called facilitated diffusion, 1D diffusion, linear diffusion, among other names) as one important target-searching mechanism [23,24]. According to this model, a sequence-specific DNA-binding protein may first interact with a DNA molecule non-specifically. Upon interaction with a non-target sequence, the protein slides along the DNA molecule until it reaches its cognate target and stops sliding. Later, 3D jumping/hopping was shown to be an alternative target-searching strategy [25,26]. The process of hopping or jumping involves the dissociation of the DNA-binding protein from the DNA molecule, its free diffusion in the 3D space, and its reassociation with a nearby DNA molecule. Theoretical studies found that in solution, 1D sliding could speed up the target search compared to 3D searching [26]. However, in eukaryotic nuclei, the presence of many DNA-binding proteins makes sliding over long distances very unlikely. Indeed, Chen et al. reported that TFs mainly employ 3D random collision as a mechanism for target search, complemented with limited 1D sliding within open chromatin regions [27]. A recent in vitro study of the zinc finger family TF GAF further revealed that GAF mainly uses 1D sliding to search for targets on open DNA (including linker DNA near nucleosomes), while it relies on 3D diffusion to bind to inner nucleosomal binding sites [28].

### 2.1. The TF Paralog-Specificity Paradox

TFs are classified into families based on their DNA-binding domains (DBDs) [29]. Common TF families include the homeodomain (HD) family [30], the basic helix–loop–helix (bHLH) family [31], the nuclear receptor family [32], etc. Each TF family (except for the zinc finger family) binds to their characteristic motifs. For example, the HD TFs bind to short AT-rich motifs [33,34], while the bHLH family binds to the so-called E-box sequences (CANNTG) [31]. In higher eukaryotes, TF families tend to have large numbers of members. These different members were generated during evolution by gene duplication followed by diversification and are thus paralogs. During evolution, more complex organisms tended to have larger TF families, which is the major supporting evidence for the idea that the increase in complexity in eukaryotes is mainly driven by more sophisticated gene regulation, rather than by higher gene numbers [35].

**Figure 2:**
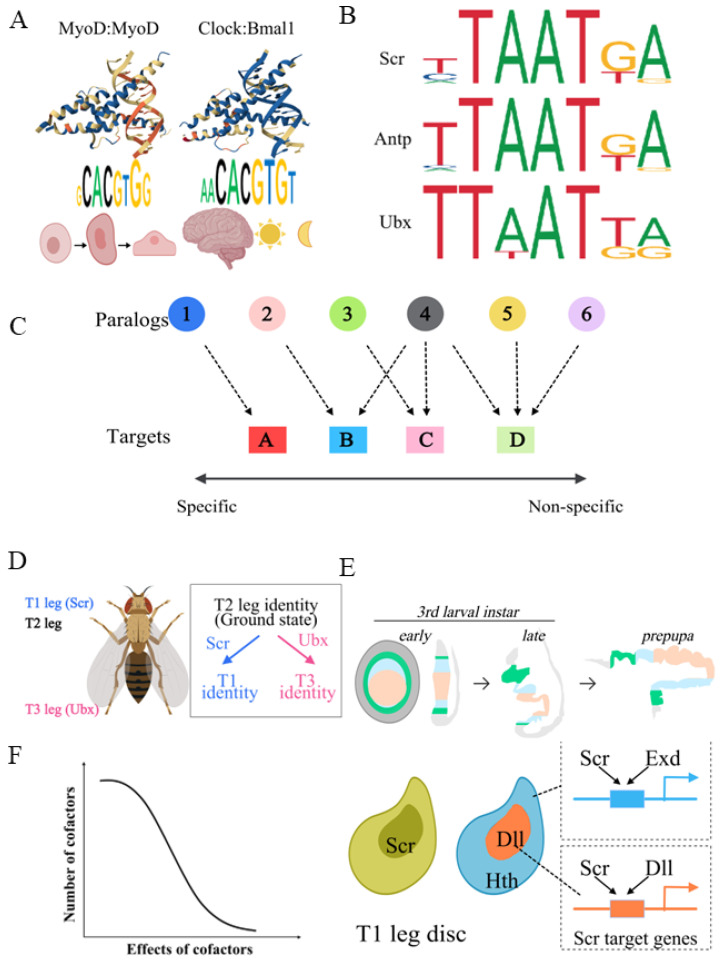
The TF paralog-specificity paradox. (**A**) Examples of different bHLH TF dimers binding to similar E-box DNA motifs but performing different in vivo functions. MyoD homodimer (wwPDB ID: 1MDY) is critical for myocyte differentiation, while Clock:Bmal1 (wwPDB ID: 4H10) heterodimer plays important roles in circadian rhythm regulation. Protein–DNA complex structures are from wwPDB [52]. (**B**) Examples of different Drosophila Hox paralogs having very similar AT-rich short binding motifs but highly distinct in vivo functions. Motifs are from JASPAR [53]. (**C**) TF targets range from most paralog specific, which are only regulated by a single paralog, to essentially non-paralog specific, which can be regulated by potentially all paralogs. (**D**) In Drosophila, Hox proteins Scr and Ubx direct differentiation of forelegs and hindlegs, respectively. (**E**) Top: Schematic showing how larval leg discs transform into adult legs during metamorphosis by evagination. Bottom: Exd and Dll are Scr cofactors in proximal and distal leg discs. Scr is expressed in entire T1 leg discs, and Hth and Dll are expressed in proximal and distal leg discs, respectively. Hth is responsible for nuclear localization of Exd. (**F**) Cofactors with large effects on TF paralog specificity are rare, and most cofactors have mild effects.

Since all paralogs of the same TF family share the same DNA-binding domain, they usually bind to very similar DNA motifs when assayed in vitro. On the other hand, they often perform highly distinct functions in vivo [36]. This so-called TF paralog-specificity paradox was first noticed during the study of Hox transcription factors in *Drosophila melanogaster* [37,38,39]. Hox TFs are one subfamily of HD TFs, and they specify segmental identity along the anterior–posterior body axis [40]. For example, in *Drosophila* the Hox TFs Scr and Ubx specify the first and third thoracic segment, respectively [41,42]. When several Hox genes were cloned, expressed in *E. coli*, and characterized for their DNA-binding properties, it was immediately noticed that all of them bound to highly similar short AT-rich motifs [43,44] (Figure 2A). Later, a similar paradox was also found in other TF families. For example, both the MyoD-MyoD homodimer and Clock-Bmal1 heterodimer belong to the bHLH family, but the former controls muscle cell differentiation, while the latter has critical functions in circadian rhythm regulation [45,46] (Figure 2B).

One mechanism to achieve paralog-specific functions is tissue-specific expression of different paralogs. If different paralogs are expressed in different tissues with distinct cellular environments (for example, neuronal cells vs. immune cells), it is not difficult to understand that they perform different functions in vivo. However, this does not fully explain this paradox. There are cases where different paralogs are expressed in homologous tissues, which have highly similar cellular environments, but show paralog-specific in vivo functions. For example, in *Drosophila*, adult legs develop from larval epithelial tissues called leg imaginal discs [47,48,49]. The three pairs of leg discs have very similar chromatin landscapes and nuclear environments, as demonstrated by results from RNA-seq, ATAC-seq and ChIP-seq experiments [42,50,51]. Nevertheless, the Hox proteins Scr and Ubx, expressed in the first and third pair of leg imaginal discs, respectively, instruct their differentiation into adult forelegs and hindlegs [41,42] (Figure 2D).

Several non-mutually exclusive mechanisms could potentially explain these phenomena. At one extreme, it is possible that multiple TF paralogs indeed bind to the same sets of target genes, but regulate each target differently. Alternatively, although different TFs have similar DNA binding motifs, these motifs are not identical. There might be some in vivo mechanisms to amplify such subtle differences to enable paralog-specific binding to a subset of target loci. It is also possible that different paralogs could bind to different downstream targets by interacting with a cofactor. It should be clarified that not all TF target loci have to be specific to a particular paralog (Figure 2C). If only one paralog is expressed in a particular tissue, its downstream targets do not need to be specific to this paralog, and it is not a problem if other TF paralogs could potentially regulate these targets. On the other hand, it is possible that a target could be regulated by several, potentially even all, paralogs. Examples include the repression of abdominal *Dll* transcription in *Drosophila* by the Hox TFs Ubx, Abd-A and Abd-B during embryogenesis [54,55], as well as the activation of the *vvl* gene expression during *Drosophila* embryogenesis by seven out of eight *Drosophila* Hox TFs [56]. Nevertheless, some TF targets are specific to a particular TF paralog [42,57,58].

The understanding of the molecular mechanisms underlying the paralog-specificity paradox have predominantly come from the study of Hox TFs. Earlier genetic and developmental studies of *Drosophila* and other model organisms identified a number of well-characterized Hox downstream target enhancers that are specific to one particular Hox paralog [57,59]. Detailed studies of these enhancers, especially the identification and mutagenesis of relevant Hox binding sites, have provided solid evidence that the Hox cofactor Exd plays important roles in Hox paralog-specific functions in vivo [59,60]. The gene *exd* was originally identified in a forward genetic screen, because its mutant does not alter hox gene expression but shows phenotypes resembling the loss of all Hox genes [61,62]. Subsequent studies elucidated that Exd forms heterodimers with all Hox proteins [43,63], and the DNA binding motifs among Exd-Hox dimers are significantly more divergent than those among Hox monomers [43]. In other words, interaction with Exd reveals the “latent DNA binding specificity” that is hidden among Hox monomers [43]. In vivo studies have also confirmed that interaction with Exd is necessary for Hox proteins to bind to and regulate some of their target enhancers [57,64]. A recent study from our group systematically compared the *Drosophila* Hox proteins Scr and Ubx in leg imaginal discs [42] (Figure 2D). This study found that over 90% of downstream targets are shared by these two paralogs, but about 8% of targets are paralog specific, i.e., only Scr or Ubx is able to bind to these targets in vivo. Functional studies have shown that interaction with Exd is necessary for Scr to bind to about half of its paralog-specific targets [42]. It is not surprising that Exd does not explain all Scr-specific binding events, because in the leg imaginal discs, Exd is nuclear in the proximal region, but resides cytoplasmically in the distal region [60,65] (Figure 2E). Therefore, Exd’s function as a Hox cofactor is restricted to the proximal leg region. On the other hand, it is well known that the distal regions of the forelegs and hindlegs show Hox-dependent morphological differences. For example, the male-specific trait sex combs are only present in the distal forelegs, thus their development is Scr-dependent but Exd-independent [66]. This suggests the existence of yet unidentified Hox cofactors in the distal leg region. A further study identified Dll as a distal region Hox cofactor that could explain about half of the Exd-independent Scr-specific binding events [42] (Figure 2E). Since we did not find evidence that Hox monomers alone are sufficient for binding to their paralog-specific targets, these results also suggest the existence of additional unidentified Hox cofactors. It is unlikely that any of these yet-to-be-defined cofactors could explain a significant number of paralog-specific binding events. Rather, it is likely that there are quite a few additional cofactors, but each of them largely works in a more restricted tissue-specific and target-specific manner [42] (Figure 2F).

A general picture from the study of Hox TFs is that cofactors are likely to play important roles in enabling paralog-specific target binding in vivo (Figure 2F). However, cofactors with large effects, such as Exd for Hox, are likely to be very rare. In fact, Exd is the only such example known so far. Most TF cofactors are likely to work in a paralog-, cell type-, and target-specific manner. This implies the existence of a significant number of potential TF cofactors with small effects, which also makes the elucidation of their in vivo functions technically challenging. It is possible that other TF families employ similar mechanisms to achieve paralog-specific functions in vivo, but how uniform these mechanisms are awaits detailed mechanistic studies on other TF families.

It must be pointed out that so far, most studies on the TF paralog-specific paradox have focused on the DNA binding step, because there are global techniques, such as ChIP-seq, DamID, Cut&Run and Cut&Tag, to characterize TF-DNA binding throughout genomes [67,68,69,70]. On the other hand, there are still no good genome-wide methods to quantify enhancer activity. There have been attempts to use histone modifications, such as H3K4me1 as markers for enhancers [71]. While this strategy has successfully identified potential enhancers, it has not revealed enhancer activity very well. The binding of factors, such as p300 and CtBP, has been suggested as indictors of enhancer activity [72], but the results are far from satisfactory in our own experiences. Therefore, although it is expected that different TF paralogs could differentially regulate a significant number of shared targets, currently we know very little about it.

### 2.2. Only a Very Small Fraction of TF Binding Sites Actually Play Regulatory Roles In Vivo

The eukaryotic genomes are very large, and transcription factors usually have a short DNA binding motif of 6–10 bp. In a diploid mammalian nucleus, a TF with a 6 bp DNA consensus binding motif could have over 1 million potential binding sites. However, when a TF is assayed with genome-wide methods, such as ChIP-seq or Cut&Tag, only thousands of targets are identified. Even considering the possibility of clustered binding sites within a single ChIP peak, these results still indicate that very few potential targets are actually bound by a TF in vivo. Moreover, most of the occupied sites are of low or intermediate binding affinities, and many high-affinity binding sites are not bound by a TF. The mechanisms of target selection are currently poorly understood, but chromatin accessibility is likely to play a major role; competition with other DNA-binding proteins is probably also involved.

In fact, for a TF, the major challenge is probably not to find and bind to the target sites it should bind to, but to avoid binding to the vast majority of sites it should not bind to. Otherwise, the promiscuous regulation of nearby genes could be detrimental to cells and organisms. One source of support for this hypothesis comes from a comparison of DNA-binding proteins between prokaryotes and eukaryotes. In prokaryotes, a target sequence is usually occupied if the corresponding DNA-binding protein is expressed. On the contrary, as stated above, this is not the case in eukaryotes. Given the fact that prokaryotic genomes are usually not longer than 10 Mbp, it is possible that this genome size represents the upper limit for when a DNA-binding transcription regulator can potentially bind to all its target sequences. For eukaryotes, the evolution of histones and other chromatin proteins permit different chromatin classes. This probably enabled the massive expansion in eukaryotic genome sizes, which again permits the evolution of the ever-increasing complexity of eukaryotic organisms.

Despite only a small fraction of binding sites being occupied by their corresponding TFs, even a smaller fraction of binding sites play regulatory roles in vivo. In other words, many TF-DNA binding events are apparently non-functional. For some time, it was assumed that intergenic and intronic regions bound by TFs were candidate enhancers. However, when DNA fragments from these regions were tested in reporter assays, they often did not show any enhancer activity. It should be pointed out that some of these fragments could function as other cis-regulatory elements, such as tethering elements or Range Extenders (see below). Even for the fragments that did show enhancer activities, when the TF binding sites were mutated, no change in enhancer activities were observed, indicating these TF binding sites were not necessary for enhancer functions. In fact, in our recent study, we estimated that only about 1/3 of the Hox-DNA binding events lead to transcription regulation [42]. Other studies have given comparable estimates [73]. A study of ER-α in a human cell line also reached a similar conclusion. In this study, it was found that despite the more than 31,000 ER-α binding sites identified genome-wide by ChIP-seq, only 1145 genes were likely to be directly upregulated by ER-α [74]. Even considering the fact that some ER-α direct targets might have multiple ER-α binding sites, this observation still suggests that most ER-α binding events are apparently non-functional.

These numbers, however, are likely to be underestimates. The reason is that the regulatory activity of DNA-bound TFs could be repressed by repressors bound nearby. For example, the embryonic salivary gland enhancer of the *Drosophila forkhead (fkh)* gene is activated by the Hox TF Scr, but this activation is repressed near the ventral midline by a yet-to-be-identified repressor [75]. Therefore, TF-DNA binding should be viewed as permissive of transcription regulation. Whether it actually leads to changes in promoter activity is also determined by other factors. From an evolutionary point of view, TF-DNA binding events must not be beneficial. Rather, non-functional TF-DNA binding events can be tolerated as long as they do not lead to promiscuous regulation that causes detrimental consequences. Furthermore, the tolerated non-functional TF-DNA binding events provide raw materials for the evolution of novel regulatory paths in the gene regulatory network (GRN).

### 2.3. Low-Affinity TF Binding Sites Play Important Functions In Vivo

**Figure 3:**
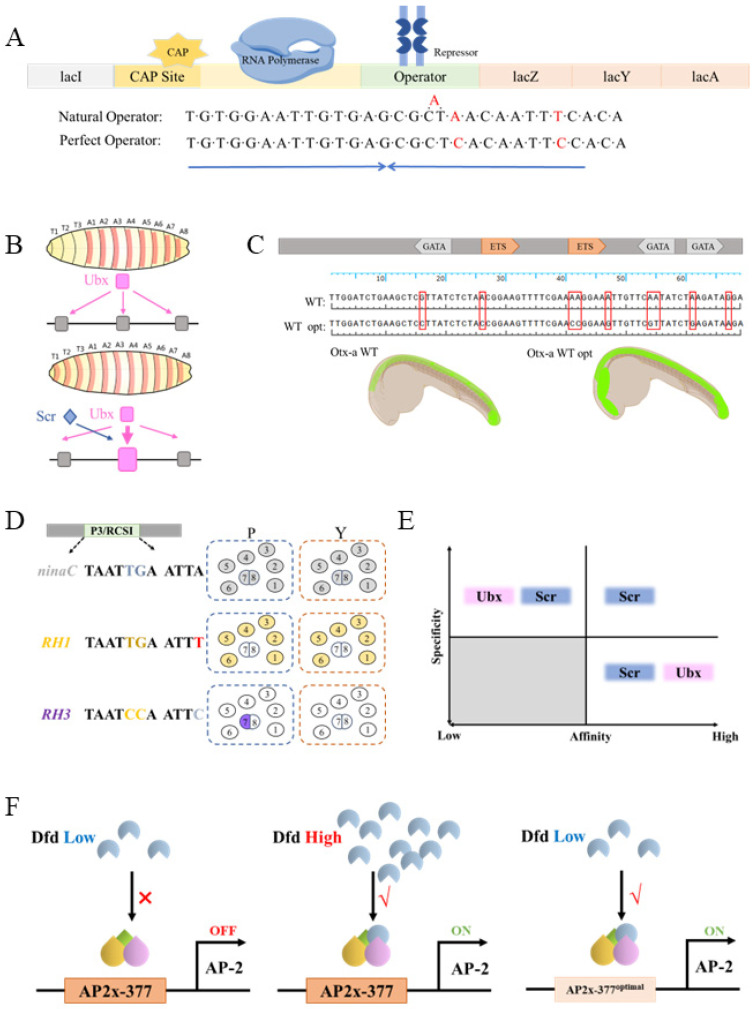
Low-affinity TF sites have important biological functions. (**A**) In *E. coli* lactose operon, the natural operator sequence is an imperfect palindromic sequence with an order of magnitude weaker repressor binding affinity than the perfect palindromic sequence. (**B**) Low-affinity Exd-Ubx binding sites ensure precise expression of the *svb* gene. When these low-affinity sites are mutated into high-affinity sites, the enhancers show ectopic expression, which depends on another Hox paralog, Scr, indicating the relaxed paralog specificity of these high-affinity sites. (**C**) In *Ciona intestinalis*, imperfect GATA and ETS target sites are necessary for a faithful *Otx-a* expression pattern. (**D**) Optimal binding sites (P3) for paired-class homeodomain TFs drive broad expression of target genes (for example, *ninaC*), while single base pair differences from the P3 sequence are responsible for the characteristic restricted expression patterns of each *rhodopsin* genes (such as *RH1* and *RH3*) in *Drosophila* photoreceptors. (**E**) The Hox protein Ubx seems to follow the classic specificity–affinity tradeoff, such that the known high-affinity Exd-Ubx sites are all less paralog specific, and all the paralog-specific Exd-Ubx sites characterized so far are all low-affinity sites. However, Scr does not obey this tradeoff strictly, and high-affinity and paralog-specific sites have been reported. (**F**) A non-canonical high-affinity Exd-Dfd site is responsible for the wild-type expression pattern of the *Drosophila AP2* gene. This site requires a high Dfd concentration to turn on target transcription. Optimization of this site to a canonical Exd-Dfd site causes responses to low Dfd concentrations, and thus ectopic reporter expression.

In biology, suboptimal DNA binding sites have repeatedly been shown to play important functions in vivo. For example, the natural *E. coli* lacO is also not a perfect palindrome, and shows a lower binding affinity for the repressor protein lacI than the artificially designed perfect palindromic lacO sequence [76] (Figure 3A). The possible biological significance of this imperfect lacO sequence is that a low leaky expression of the three genes in the lac operon is necessary for quick responses to lactose in media, and is therefore important for fitness.

Low-affinity binding sites are also prevalent in eukaryotes [36]. When studying the regulation of the shavenbaby (svb) gene by the Hox TF Ubx in *Drosophila* embryogenesis, it was discovered that some functionally important Ubx downstream target enhancers contained several low-affinity Exd-Ubx binding sites [77]. When several low-affinity binding sites were replaced with a single high-affinity Exd-Ubx binding site, the Ubx target enhancer showed ectopic activity, which depended on the expression of other Hox paralogs [77] (Figure 3B). Therefore, a tradeoff between binding affinity and paralog specificity was proposed. According to this model, high-affinity TF binding sites are less selective among different TF paralogs, whereas highly paralog-specific TF target sites cannot have high DNA-binding affinity (Figure 3E). More case studies have supported the above paradigm. When analyzing the *Otx-a* enhancer in the invertebrate chordate *Ciona intestinalis*, it was discovered that the wild-type GATA and ETS target sites were suboptimal and low affinity. Replacing them with high-affinity consensus binding motifs also caused ectopic expression [78] (Figure 3C). Another study found that perfect and imperfect binding sites on the same TFs had evolved to drive the expression of genes with broad expression patterns vs. more restricted expression patterns. In *Drosophila* photoreceptor cells, phototransduction genes and rhodopsin genes are both activated by paired-class homeodomain TFs, but the phototransduction genes are broadly expressed, while each of the rhodopsin genes is only expressed in a subset of cells [79]. Significantly, the enhancers of the phototransduction genes contain perfect activator binding sites, whereas the enhancers of the different rhodopsin genes each contain a unique 1 bp difference from the perfect binding site. These 1 bp substitutions are sufficient to drive each rhodopsin gene to be expressed in different subsets of the photoreceptor cells [79] (Figure 3D).

Research on other Hox paralogs, however, has indicated a more complex picture. In our recent work, we identified and functionally characterized dozens of Scr-specific target enhancers in leg imaginal discs [42]. We were able to identify the critical Exd-Scr target sites within some of them. Many of these Exd-Scr sites show strong selectivity for Scr compared to other Hox paralogs. Although some of them are low affinity, we did find a few high-affinity sites that are nevertheless very Scr specific [42] (Figure 3E). In another study, analysis of the *Drosophila* AP-2 enhancer in embryos also identified binding sites very specific to the Exd-Dfd heterodimer. Further analysis revealed that although this site is a non-canonical Exd-Dfd site, its affinity to Exd-Dfd is quite high. Nevertheless, optimization of this site to a canonical site causes ectopic reporter expression [58] (Figure 3F). While the affinity–selectivity tradeoff likely needs to be modified from its simplistic original version [77], it is clear that low-affinity TF binding sites play important functions in vivo under many different cellular contexts.

The prevalence of low-affinity binding sites presents a challenge for TFs to efficiently bind to DNA. To ensure the fraction of TF-bound enhancer is high enough to sustain transcription regulation, a high TF concentration is required if the enhancer contains low-affinity binding sites. However, TFs are usually not high-expression proteins, and the number of TF molecules in a single nucleus can be limited. Estimates range from hundreds to thousands of TF molecules per nucleus, which is usually not sufficient to achieve significant enhancer occupancy. Readers are referred to Kribelbauer et al. [36] for an excellent discussion of the relationship between TF concentration and enhancer occupancy. The solution to this puzzle is that TFs do not have a uniform concentration in nuclei. Instead, imaging experiments have showed that TFs often form discrete hubs with high local concentrations [80]. In addition, enhancers often contain multiple low-affinity binding sites [77,78]. High local concentrations of TFs and their binding sites, as well as those cooperativity created by interactions among the TFs and other DNA-binding proteins occupying the same enhancer, are believed to be the driving force that ensures sufficient TF-DNA binding at enhancers.

### 2.4. TF-DNA Binding in Eukaryotes Is Highly Dynamic

Early attempts to study the dynamics of protein–DNA binding in vivo mainly relied on Fluorescence Recovery After Photobleaching (FRAP) assays to measure fluorescence photobleaching recovery kinetics [81]. To perform a FRAP, the DNA-binding protein being tested is fluorescently labeled, and a selected region of the nucleus is photobleached using a high-power laser beam. The rate of fluorescent recovery is then measured and quantified. This rate depends on the diffusion rate of the labeled DNA-binding protein in the nucleus, the dissociation rate of the photobleached protein molecules, and the association rate of the fluorescent protein diffused into the photobleached region. Early studies established that, in general, histone–DNA binding is relatively stable, with the residence time in the range of hours [82]. However, when the fluorescently labeled sequence-specific TFs were photobleached, the fluorescence recovery showed very fast dynamics, indicating that TFs generally have short residence times on DNA [83,84,85].

Precise measurements of TF-DNA binding kinetics have become possible in the past two decades with the development single-molecule tracking (SMT) technology [86]. By tracking the movements of hundreds of fluorescently labeled single TF molecules and applying sophisticated statistical tools, several important observations have emerged. Only a small fraction of all TF molecules were found to bind to DNA [87], as low as 3% for the TF Sox2 [27], while the majority were found to be freely diffusing. Thus, TFs spend most of their time searching for their cognate binding targets. Contrary to the conventional model that posits that once TFs bind to enhancers they stay on the DNA to regulate transcription, the SMT experiments showed that most TF-DNA binding is very dynamic, with a residence time in the range of 10s of seconds when bound to cognate binding sites [87].

From an evolutionary point of view, highly dynamic TF-DNA interactions enable quick responses to internal and external regulatory signals. If TF-DNA binding featured long residence times and slow turnover rates, transcription responses would be very slow if the responses required the bound TFs to dissociate from DNA. Or, there would have to be active mechanisms to remove the bound TFs, which could be energetically costly. This advantage is even more significant when taking into account the fact that in reality, many physiological responses to internal and external signals involve multiple levels of transcription regulation. The primary responses might involve changes in the expression of some TFs, which in turn regulate their downstream targets, many of which might again be TFs. Highly dynamic TF-DNA interactions let organisms to employ a combination of direct and indirect transcription responses to react to various cues.

## 3. The Mechanisms by Which TFs Regulate Promoter Activities

TFs receive and integrate various biological inputs, and collectively determine the biological responses in the form of transcription patterns and levels. TF–enhancer binding is the first step in TF-mediated transcription regulation. After binding to DNA, TFs must somehow influence transcription activities at the promoters. The traditional notion was that TFs mainly affect transcription initiation. Recent advances have indicated that this was an oversimplification. For the transcription of many eukaryotic genes, RNA pol II pauses after transcribing 50–70 nt of the nascent RNA [3,88]. Further, it has been shown that the release of paused RNA pol II represents a very important transcription regulatory point (Figure 1A). Recent data have indicated that TFs also regulate the rate at which paused RNA pol II is released [6]. Regardless of whether a TF regulates initiation or pause release, it must somehow transduce its regulatory signal from the enhancer to the promoter. Adding a layer of complexity to the picture, eukaryotic transcription does not proceed at a constant rate. Rather, live imaging studies have revealed that transcription is stochastic and happens in discontinuous bursts [89]. TFs achieve transcription regulation by influencing parameters like burst frequency and burst amplitude. The stochastic nature of transcription is one of the mechanisms underlying transcriptional noise. How cells achieve robust regulatory outcomes from the noisy nature of transcription is still not well understood [90]. In this section, we review and discuss the molecular mechanisms of how TFs regulate RNA pol II initiation and pause-releasing rates.

### 3.1. Eukaryotic Transcription Requires Multiple TF-DNA Binding Events at Enhancers

**Figure 4:**
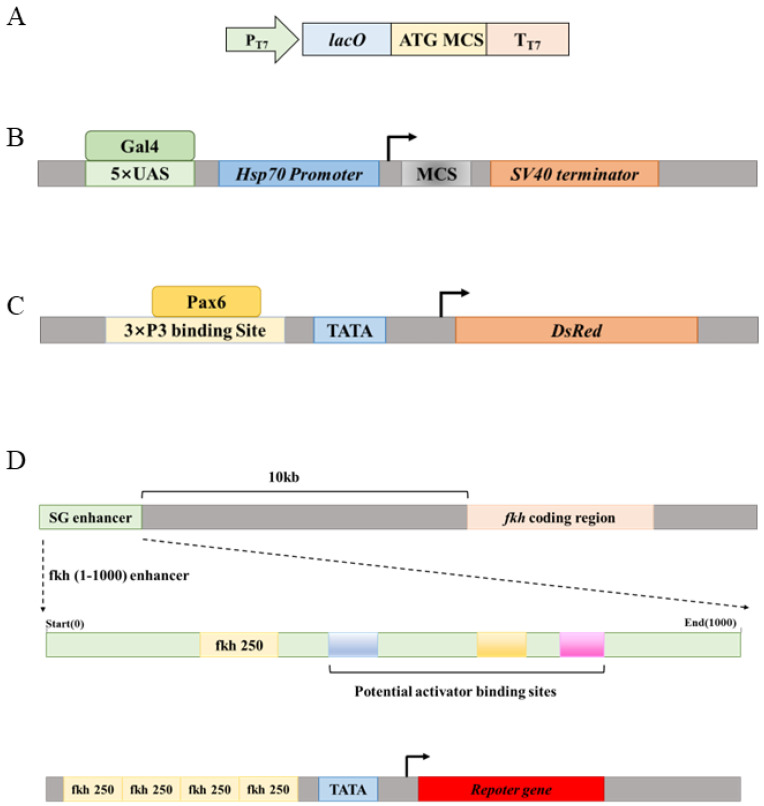
Eukaryotic transcription requires multiple TF-DNA binding events at enhancers. (**A**) Schematic showing lacO site in some versions of pET vectors as an example that, in prokaryotes, a single TF-DNA binding event is often sufficient for function. (**B**) Schematic of the pUAST family of vectors commonly used in the *Drosophila* community. Note that at least 5 copies of the UAS sequence are necessary for Gal4-induced transcription. (**C**) Schematic of the *3xP3-DsRed* reporter commonly used as a marker for transgenesis in various insect species. Three copies of the eye-specific Pax6 TF binding site are used. (**D**) Top: Schematic of the salivary gland (SG) enhancer of the *Drosophila forkhead (fkh)* gene. The fkh250 site is absolutely necessary for enhancer function, but several yet-to-be well-characterized activator binding sites scattered across the entire enhancer quantitatively boost the transcription level. Bottom: When the necessary fkh250 site is used alone to drive reporter expression, concatemers of 4 copies of the site are used, and the expression levels are still quite low.

In prokaryotes, there are many cases where a single TF binding site is sufficient for regulatory functions. For example, in many versions of the pET series of expression vectors, one lacO site is sufficient to confer lacI-mediated repression, which is lifted by the commonly used inducer IPTG [91] (Figure 4A). However, in eukaryotes, abundant evidence indicates that a single TF binding site is not sufficient to form a functional enhancer. Rather, any functional enhancer must be bound by multiple identical or different DNA-binding proteins, forming a complex called the enhanceosome. For example, yeast Gal4 is one of the best understood transcription factors. It binds to the UAS sequence and drives the transcription of its target genes. The Gal4-UAS system has been used extensively in *Drosophila* to drive heterologous gene expression, but a single copy of UAS is not sufficient to achieve significant Gal4-dependent gene expression. To induce robust Gal4-dependent gene expression, usually at least five copies of the UAS sequence is necessary [92,93] (Figure 4B). Similarly, the 3xP3 enhancer is a popular marker for transgenesis in various insect species, which drives robust reporter gene (often EGFP or DsRed) expression in insect eyes. This enhancer contains three copies of consensus Pax6 binding sites to ensure robust reporter expression [94] (Figure 4C).

A more convincing case has come from the study of the embryonic salivary gland enhancer of the *Drosophila forkhead (fkh)* gene (Figure 4D). Enhancer-bashing experiments identified a 1000 bp salivary gland-specific enhancer about 10 kb upstream of the fkh coding region [75]. This enhancer, named the fkh(1–1000) enhancer, drives robust reporter expression in embryonic salivary gland placodes, where the fkh gene is normally expressed [95]. An Exd-Scr heterodimer binding site was identified at the 250 bp position (therefore named the fkh250 site) that is necessary for the normal function of the fkh(1–1000) enhancer. However, in order to use the fkh250 site alone as an enhancer to drive reporter expression, it requires a concatemer of four copies of a 37 bp DNA fragment containing the fkh250 site [57]. Even so, the reporter expression level is still significantly weaker than that for the fkh(1–1000) enhancer. Moreover, systematic enhancer truncation experiments showed that the first 506 bp and the first 308 bp of the fkh(1–1000) enhancer still drive reporter expression in embryonic salivary gland placodes, but as the DNA fragment becomes shorter and shorter, the reporter expression level becomes weaker and weaker [75]. These results suggest that there are proteins bound in the 309–505 bp and 507–1000 bp regions, which are not necessary for salivary gland placode expression but nevertheless increase reporter expression in an apparently additive manner. Indeed, systematic mutational studies of enhancers have revealed that the majority of mutations alter enhancer activities to some extent, indicating that the density of functional TF binding sites within enhancers are quite high [96,97].

The above cases, as well as others, indicate that there seems to be a threshold or “critical mass” of enhancer-bound proteins needed to form a functional enhanceosome. Some studies have indicated that the formation of enhanceosomes is semi-hierarchical, meaning that some proteins must bind to the enhancer before recruiting others [87]. As illustrated in the above case of the fkh(1–1000) enhancer, different enhancer-bound TFs show different levels of effects on transcription outcomes. Some are absolutely necessary for enhancer function, while others apparently play more quantitative roles to ensure robust and strong gene transcription.

### 3.2. The Mediator Complex Bridges the Enhancer and the Promoter

When an enhanceosome is formed at an enhancer, it must somehow convert all biological inputs integrated at the enhancer into physiological responses in the form of transcription patterns and levels. The Mediator complex plays an important role in this process [9,10]. The discovery of the Mediator complex was a result of the synergy between genetic and biochemical explorations of transcription mechanisms. Mediator subunits were identified as mutants affecting global transcription in yeast, and as protein subunits biochemically purified as complexes interacting with RNA pol II or TF activation domains [98,99]. In all reported cases so far, knock-out of individual Mediator subunits in mice resulted in embryonic lethality [10,100]. Yeast seems to tolerate mutations in Mediator complex subunits better—the individual knock-out of 10 out of 25 Mediator subunits shows lethality [100]. It is now well established that the Mediator complex is necessary for the transcription of all protein-coding genes.

The Mediator complex is a very large multi-subunit complex organized into four separate modules: the head, middle, tail modules and a kinase module [9,10,100]. The head and middle modules interact with RNA pol II and GTFs, and they stabilize the PIC. The tail module mainly interacts with enhancer-bound TFs and transmits regulatory signals to the promoter. The kinase module can associate with or dissociate from the other modules in a reversable manner, and plays important regulatory roles in transcription. The yeast Mediator has about 25 different subunits, and the human version has roughly 29. The composition of the Mediator complex shows some context-dependent flexibility [9,10,100].

The subunits of the Mediator complex do not directly bind to DNA in a sequence-specific manner. Rather, they are recruited to enhancers and promoters via protein–protein interactions with DNA-binding proteins. Different TFs have been shown to physically interact with different Mediator subunits. For example, Med1 seems to be a common target for nuclear receptors [101], while Med23 interacts with TFs like ELK-1 and E1A [102]. The interaction between TFs and Mediator subunits shows some degree of promiscuity, such that one TF might interact with several Mediator subunits. For example, both Med23 and Med31 are required for transcription activation induced by heat shock factors [103]. One possible reason for the requirement of multiple proteins on an enhancer is that it takes multiple protein–protein interactions for the enhancer to efficiently recruit the Mediator complex. These protein–protein interactions likely involve different TFs that interact with different subunits of the Mediator complex.

### 3.3. Chromatin Often Forms Loops Between Enhancers and Promoters

To transmit the regulatory signals communicated by enhancer-bound TFs to transcription responses at the promoters, the Mediator complex interacts with both the proteins bound at the enhancer and those bound at the promoter. Therefore, it is expected that the TF-occupied enhancer and the promoter it regulates are in close physical proximity in the nucleus. In higher eukaryotes, an enhancer could be hundreds of kbs away from the promoter it regulates. It was therefore proposed that long DNA molecules, where both the promoter and the enhancer reside, form a loop to ensure that the enhancer and the promoter are close to each other. Supporting this assumption, Hi-C experiments have shown repeatedly that, in many cases, the enhancer and the promoter it regulates are indeed in close physical proximity [104,105].

In fact, DNA loops are prevalent in eukaryotic nuclei. It was proposed that loops first evolved to deal with the chromatin packaging challenge faced by all eukaryotic organisms, and were later co-opted to gene regulation [106]. Loops not only bring together enhancers and promoters, but they are also necessary for the formation of higher-order chromatin structures, such as TADs (topologically associated domains) [107,108,109]. The molecular mechanisms of loop formation have been elucidated relatively well. Loop extrusion is the main mechanism of loop formation [110], and proteins like CTCF and Cohesin are necessary for the proper formation of DNA loops [111].

**Figure 5:**
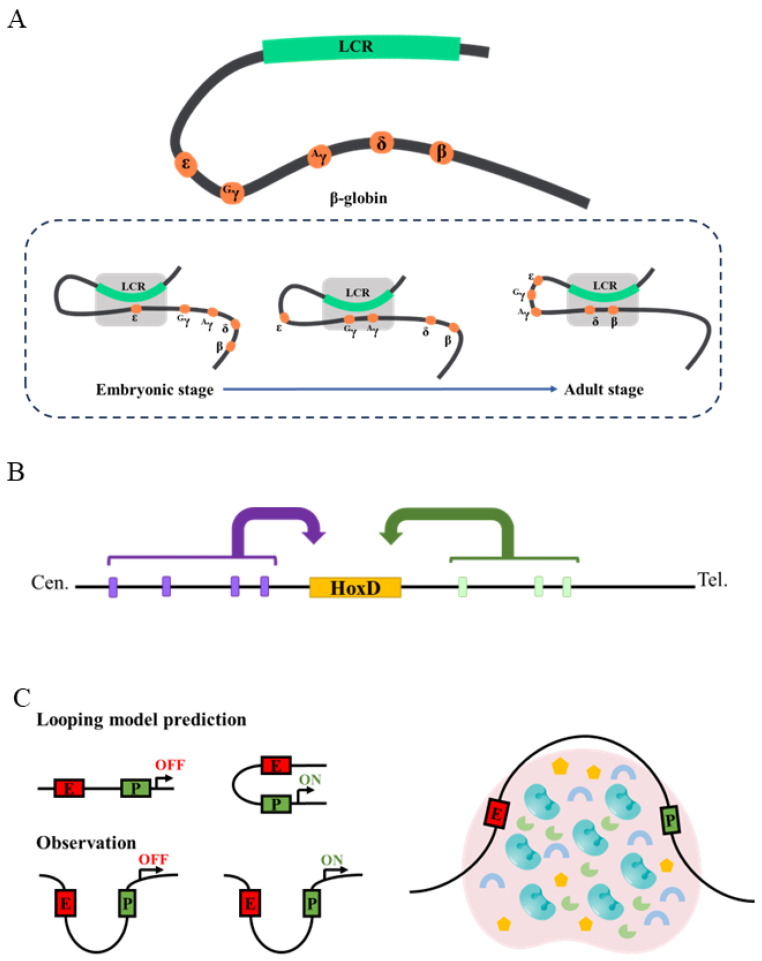
The mechanisms by which enhancers regulate promoters. (**A**) In a mammalian β-globin locus, the enhancer LCR is brought into close proximity to the particular globin gene it activates. As development progresses, different globin genes are expressed, and the LCR is always looped to the expressed globin genes. (**B**) A mammalian HoxD locus displays a stable looping conformation regardless of whether the promoter is active or not. (**C**) Left: The prediction of the looping model vs. the observed results. Right: The condensate model of enhancer–promoter communication.

The relationship between enhancer–promoter loops and transcription regulation is multifold. There are well-known cases in which the loops between enhancers and promoters are necessary or near necessary for proper transcription. For example, many genes expressed in *Drosophila* embryos show loops between their enhancers and promoters a few hours before these genes are turned on transcriptionally [112]. Similarly, the *β-globin* locus has an enhancer (Locus Control Region, or LCR) that controls the expression of different globin genes at different developmental stages [113]. 3C experiments showed that before the expression of each globin gene, the LCR forms a loop with the corresponding promoter [114] (Figure 5A). Significantly, disrupting the loops by knocking out the looping factors reduces globin gene expression, while creating an artificial loop by tethering the looping factors leads to ectopic globin expression [115,116]. These results suggest that loop formation between the LCR and globin gene promoters is both sufficient and necessary for LCR-activated transcription of globin genes. The mouse HoxD locus also contains various loops that bring together enhancers and the promoters they regulate [104,117]. Interestingly, while the *β-globin* locus displays dynamic looping conformation throughout development, the *HoxD* locus shows an apparent stable looping conformation across different tissue types, such that the loop conformation is similar regardless of whether the promoter is active or not (Figure 5B).

### 3.4. The Condensate Model of TF Action

Although the loop model explains many experimental results very well, in the past decade, there have been several sets of results contradictory to the loop model. First, in *Drosophila* embryos, when a single enhancer was placed between two promoters whose transcription directions were away from the enhancer in the middle, live imaging of nascent RNA produced from these two promoters showed that they burst in a more or less synchronous pattern [89]. If the classic looping model, in which one enhancer contacts one promoter at a time, was correct, the two promoters should not have fired at the same time because the enhancer in the middle could only loop to one of the two promoters at any single time. More significant counter examples have come from imaging experiments [118,119,120]. In these experiments, arrays of different protein binding sequences (such as lacO and cuO) were inserted in close vicinity to an enhancer and promoter, and the corresponding binding proteins (lacI and CymR, respectively) were fused to different fluorescent proteins. This setup enabled the visualization of both the enhancer and the promoter in the nucleus, and their distances were quantified. Various such imaging experiments on different genes have showed that when a promoter is actively transcribing nascent RNA, its enhancer can be far away (200–300 nm), excluding direct enhancer–promoter contact [118] (Figure 5C). Moreover, in many cases the enhancer–promoter distance is even larger when a gene is turned on than when it is turned off [118,121]. One possibility for this is that there exists an activation phase and a self-maintenance phase in enhancer-driven promoter activation. The activation phase must be initiated by direct enhancer–promoter contact, but once the promoter is activated, there are mechanisms that self-sustain promoter activity, at least for some time. This model has recently received some experimental support [118], but how general it is remains to be seen.

Currently, a popular model is the condensate model of transcription regulation. According to this model, proteins, such as RNA pol II, GTFs, the Mediator complex, and sequence-specific TFs, organize into structures called condensates that have sizes in the range of 100–1000 nm [122]. The presence of condensates has been confirmed by imaging-based studies [89,123,124]. Condensate formation is a major mechanism leading to the non-uniform distribution of TFs and other factors in nuclei. Condensates facilitate enhancer-driven promoter activation by at least two mechanisms (Figure 5C). Condensates can embed both enhancers and promoters, and the various proteins necessary for transcription have much higher concentrations in condensates. There is ample evidence that condensate-forming proteins often have intrinsically disordered domains (IDRs), also called low-complexity domains [125]. Contrary to the stable interactions mediated by structured protein domains, IDRs have been shown to mediate weak multivalent interactions that drive the formation of condensates [122,125,126]. While there are still controversies about whether condensates truly represent liquid–liquid phase separation (LLPS) [126], it is clear that condensates increase local concentrations of various factors.

The formation of condensates might be initiated by protein–DNA binding [127]. When multiple proteins bind to enhancers and promoters, they form the seeds to recruit more factors via dynamic multivalent interactions mediated by their IDRs. IDR-mediated interactions are selective, so condensates can enrich some factors while excluding others [128]. The result is that different condensates have different components, and therefore different properties. Condensate formation can also be initiated solely off of DNA by protein–protein interactions [123]. When IDRs are deleted, condensate formation and transcription are often both affected. In addition, certain chemicals are found to disrupt condensates in vitro and in vivo, presumably by disrupting the weak multivalent interactions among IDRs. Treating cells with these chemicals also affects transcription [129]. These results strongly support the significance of condensates in transcription regulation.

Interestingly, sometimes it seems that both DNA loops and transcription condensates contribute to transcription regulation. For example, Hi-C experiments showed that the Sox2 enhancer and promoter interact with each other [130]. On the other hand, imaging results showed that these two loci are often hundreds of nanometers apart in the nucleoplasm [118]. The discrepancy between the imaging and Hi-C can be explained by the fact that only a very small portion of enhancer–promoter pairs are close enough to be captured by Hi-C, and most of the time they are far away. Indeed, a study of nuclear receptor ERα showed that enhancer–promoter contact frequency increases upon transcription activation, but their physical proximity is decreased [131]. This highlights the importance of using a combination of different techniques to obtain a full picture of transcription regulation.

### 3.5. New Models Are Needed to Fully Explain How TFs Regulate Promoters

The condensate model could explain many of the abovementioned experimental results. Nevertheless, it needs to be significantly refined and elaborated. For example, recent results showed that RNA pol II-containing condensates may not be associated with either enhancers or promoters, but they could still somehow boost promoter firing [123]. In addition, even without any detectable association with RNA pol II-containing condensates, many promoters still fire, albeit at a lower rate [123]. Another issue the condensate model has difficultly explaining is how insulators block enhancer–promoter communication. Insulators are DNA elements that, when placed between an enhancer and a promoter, usually block the regulation of the promoter by the enhancer [132]. Condensates are 3D structures, and it is difficult to envision how their functions are blocked by insulators. Similarly, it is known that enhancers and promoters must generally reside in the same TAD [107,108,109]. It is not obvious why the functions of condensates are blocked by TAD boundaries.

We propose that one possible route to refine the condensate model is to analyze protein–protein interactions in detail. After all, enhancer-driven promoter activation must be driven by series of protein–protein interactions. The presence of condensates and the roles of IDRs are clear; what is missing are the details of IDR interactions and interactions involving structured domains. In particular, it is known that IDR interactions have specificity, but the nature of their specificity is complicated and poorly understood. Elucidating the protein–protein interactions in detail not only holds promise for refining the condensate model, but also for understanding the mechanisms of IDR interaction specificity, which underlies condensates selectively.

## 4. The Molecular Mechanisms of Enhancer–Promoter Matching

The above discussions focused on how an enhancer regulates its matching promoter. However, how an enhancer correctly identifies and locates its matching promoter in a crowded nucleoplasm is not a simple task. Although eukaryotes generally do not have significantly more than 30,000 genes, many genes have multiple promoters [133]. The number of potential enhancers can be 1–2 orders of magnitude more than the promoters [71]. Finding a match in such a situation is an enormous task. For example, the *Drosophila BX-C* complex has three *Hox* genes, *Ubx*, *abd-A* and *Abd-B*. They have one, three and six annotated promoters, and are regulated by four, three and four epithelial enhancers (as well as other non-epithelial enhancers) that collectively drive their expression in stereotyped patterns along the anterior–posterior body axis [134,135,136]. Moreover, an enhancer may not regulate the nearest promoter at the DNA sequence level. For example, the *iab-5* enhancer regulates *Abd-B*, but it is closer to the *abd-A* promoters [134,135,136].

Similarly to the TF-DNA binding task, the challenge any enhancer faces is not only to find and regulate its matching promoter, but also (and probably even more importantly) to avoid mis-regulating promoters it should not regulate. This becomes an even bigger challenge when considering the fact that there is no apparent enhancer–promoter incompatibility. In other words, it seems that any enhancer has the potential to pair with and regulate any promoter. For example, enhancers are often functionally tested using reporter assays [93]. In these experiments, generally only a few promoters are used, but it seems that the enhancers tested have little issue matching with these promoters, although an enhancer might show quantitative differences in its activity when paired with different promoters [93]. Nevertheless, in vivo, enhancers and promoters must be tightly matched. The mechanisms underlying enhancer–promoter matching are currently poorly understood.

Several mechanisms might explain how enhancers match with their promoters. One model is based on pre-formed loops. Indeed, a class of elements named the “tethering elements” have been identified near enhancers and promoters [137,138]. They do not display any enhancer or promoter activities, but help in forming loops between enhancers and promoters. When the tethering elements are deleted, the loops disappear and transcription is affected [137,138]. The recently reported Range Extender (REX) elements have similar functions, but are only necessary for long-distance looping between an enhancer and its promoter, while they are dispensable for short-distance enhancer–promoter matching [139]. These elements are likely bound by the proteins involved in loop formation. This model, however, does not really solve the enhancer–promoter matching puzzle. There are potentially many tethering elements and REX elements in the genome, so the question now becomes, how are tethering elements correctly paired?

The multivalent interactions among DNA-binding proteins at enhancers and promoters can nucleate dynamic, condensate-like assemblies that concentrate Mediator, Pol II, and other regulatory factors. These transient hubs could act as kinetic scaffolds that enhance Pol II recruitment and increase the probability of productive enhancer–promoter contacts without requiring stable looping [122]. This model is possible, but detailed characterizations of the hubs are necessary to provide supporting evidence. It is also difficult to imagine that the chemical properties of the large number of potential nuclear condensates could be diverse enough to match all enhancer–promoter pairs. Therefore, it is more likely that the condensate model represents one of many mechanisms that ensures correct enhancer–promoter matching.

## 5. Conclusions

Over the nearly six decades since the initial discovery of eukaryotic RNA polymerases in 1969 [1], research has established the fundamental principles of eukaryotic transcription. On the other hand, new discoveries are being made every day. Yet, major questions remain unresolved, particularly concerning the mechanisms underlying TF actions, paralog specificity, condensate organization, and enhancer–promoter matching. Future progress will depend on integrating genomics, imaging, biochemistry, and quantitative modeling to dissect protein–protein interactions at high resolutions. A mechanistic understanding of these processes will not only refine current models but also illuminate how transcriptional regulation evolves and operates in complex organisms.

## Data Availability

No new data were created or analyzed in this study. Data sharing is not applicable to this article.
